# News and Notes

**Published:** 1911-06

**Authors:** 


### NEWS *and* NOTES

### MEDICAL EDITORS TO MEET IN LOS ANGELES, CAL.

The 42nd Annual Meeting of the American Medical Editors' Association, will be held at the Alexandria Hotel, Los Angeles, on June 26th and 27th, under the Presidency of Dr. J. Mc-

Donald, Jr., of N. Y. with the Annual Banquet on the evening of Monday, June 26th, at the above Hotel.

Among the papers to be presented at this meeting are the following, all of which are of interest to Medical Editors, and all doctors who are journalistically interested are invited to attend this session.

"Relation of the Medical Press to the Public Health and Marine Hospital Service," by Walter Wyman, Surgeon General; "The Advisability of Newspapers and Magazines Having Medical Editors on their Staff," by Edgar A. Vander Veer, M.D.; "Some Things I have Learned as a Western Medical Editor," by Edward C. Hill, M.D.; "Some Elements of Success in Medical Journalism," by J. M. French, M.D.; "The Medical Reporter from his Own Standpoint," by E. Franklin Smith, M.D.; "Physical Therapeutics in the Medical Press," by Arnold Snow, M.D.; "What Shall We Publish," by J. R. Phelan, M.D.; "The Extension of Advertising in Medical Journals," by S. DeWitt Clough; "Medical Expert Testimony," by R. B. H. Gradwohl, M.D.; "The Hospital Bulletin as a Factor in Medical Journalism," by George W. Kosmac, M.D.; "The Literary Side of Medical Journalism," by T. D. Crothers, M.D.; "Private Owned Medical Journals," by Henry W. Coe, M.D.; "The Influence of Medical Journalism for Medical Progress," by W. Benham Snow, M.D.; "Editorial Independence," by T. G. Atkinson, M.D.; Subject to be announced, by C. H. Hughes; Subject to be announced, by William Porter, M.D.

---

### ARMY EXAMINATIONS.

The Surgeon General of the Army announces that preliminary examinations for the appointment of first lieutenants in the Army Medical Corps will be held on July 10, 1911, and September 5, 1911, at points to be hereafter designated.

Full information concerning these examinations can be procured upon application to the "Surgeon General, U. S. Army, Washington, D. C." The essential requirements to securing an invitation are that the applicant shall be a citizen of the United States, shall be between 22 and 30 years of age, a graduate of a medical school legally authorized to confer the degree of doctor of medicine, shall be of good morau character and habits, and shall have had at least one year's hospital training, after graduation. The examinations will be held concurrently throughout the country at points where boards can be convened. Due consideration will be given to localities from which applications are received, in order to lessen the traveling expenses of applicants as much as possible.

The examination in subjects of general education (mathematics, geography, history, general literature and, Latin) may be omitted in the case of applicants holding diplomas from reputable literary or scientific colleges, normal schools or high schools, or graduates of medical schools which require an entrance examination satisfactory to the faculty of the Army Medical School.

In order to perfect all necessary arrangements for examination, applications must be complete and in possesion of the Adjutant General at least three weeks before the date of examination. Early attention is therefore enjoined upon all intending applicants. There are at present sixty-one vacancies n the Medical Corps of the Army.

